# Suppression of Retinal Neovascularisation by Tetrahedral Framework Nucleic Acids‐Resveratrol Via Dual Anti‐Angiogenesis and Anti‐Inflammation

**DOI:** 10.1111/cpr.70227

**Published:** 2026-06-03

**Authors:** Yili Jin, Tao Cai, Junyang Huang, Li Chen, Yi Lin, Jingying Liu, You Wang, Xiaoyan Ding

**Affiliations:** ^1^ Department of Ophthalmology, Sir Run Run Shaw Hospital Zhejiang University School of Medicine Hangzhou China; ^2^ Sichuan Eye Medical Center, School of Medicine University of Electronic Science and Technology of China Chengdu China; ^3^ Sichuan Eye Medical Center, Sichuan Provincial People's Hospital University of Electronic Science and Technology of China Chengdu China; ^4^ The First School of Clinical Medicine Southern Medical University Guangzhou China; ^5^ Department of Ophthalmology, General Hospital of Central Theater Command Wuhan China

**Keywords:** anti‐angiogenesis, anti‐inflammation, MAPK pathway, resveratrol, retinal neurovascular unit, tetrahedral framework nucleic acids

## Abstract

Retinal neovascular diseases, such as retinopathy of prematurity and diabetic retinopathy, threaten vision by disrupting retinal structure and function through pathological neovascularisation and chronic inflammation. Existing anti‐VEGF therapies primarily target angiogenesis, offering limited control over inflammation and requiring repeated administration. Here, we developed a resveratrol (RSV)‐loaded tetrahedral framework nucleic acid nanostructure (tFNAs‐RSV) to achieve concurrent anti‐angiogenic and anti‐inflammatory effects. tFNAs provided a biocompatible, stable and penetration‐efficient carrier that enhanced RSV ocular delivery and bioactivity. In hypoxia‐induced retinal neovascularisation models, tFNAs‐RSV markedly reduced neovascular lesion area, vascular leakage and retinal inflammatory cell infiltration. Mechanistically, tFNAs‐RSV suppressed HIF‐1 signalling and inhibited the p38 MAPK pathway and NF‐κB p65 pathway. Collectively, these results validate tFNAs‐RSV as a promising nanoplatform for treating retinal neovascular diseases, with an emphasis on inhibiting neovascularisation and inflammation.

## Graphical abstract

Resveratrol‐loaded tetrahedral framework nucleic acids (tFNAs‐RSV) enable concurrent anti‐angiogenic and anti‐inflammatory therapy, enhancing ocular delivery and bioactivity. In hypoxia‐induced retinal neovascularisation, tFNAs‐RSV reduce neovascular lesions, vascular leakage and inflammatory infiltration by suppressing HIF‐1, p38 MAPK and NF‐κB p65 signalling.

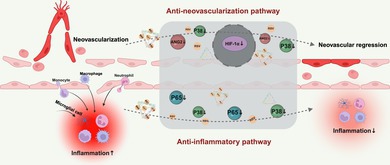

## 1 Introduction

Retinal neovascular diseases, including retinopathy of prematurity, proliferative diabetic retinopathy and retinal vein occlusion [1], are characterised by a multifaceted pathological process stemming from an imbalance between pro‐angiogenic and anti‐angiogenic factors in the retina [2, 3, 4, 5]. In these conditions, retinal hypoxia triggers excessive angiogenesis by inducing pro‐angiogenic mediators such as vascular endothelial growth factor (VEGF), while simultaneously activating inflammatory cascades—most notably NF‐κB signalling—that drive cytokine release, leukocyte infiltration and breakdown of the blood–retinal barrier. The interplay between uncontrolled vessel growth and chronic inflammation exacerbates vascular leakage and accelerates retinal damage. Current anti‐VEGF therapies effectively suppress angiogenesis but have limited impact on inflammation [6], while anti‐inflammatory agents alone fail to halt pathological vessel growth. These shortcomings highlight the urgent need for novel therapeutics capable of concurrently suppressing aberrant angiogenesis and inflammation to achieve more effective and durable disease control [7].

Resveratrol (RSV), a naturally occurring polyphenolic compound found in grapes, berries and peanuts, has emerged as a promising therapeutic candidate for conditions where angiogenesis and inflammation are closely intertwined. It possesses a broad spectrum of bioactivities, including anti‐inflammatory, anti‐angiogenic, antioxidant and neuroprotective properties. In ocular models, RSV has been shown to inhibit pathological neovascularisation by suppressing VEGF expression and interfering with pro‐angiogenic signalling pathways. Concurrently, it attenuates inflammatory responses by reducing phosphorylation and nuclear translocation of NF‐κB p65, thereby down‐regulating the expression of pro‐inflammatory cytokines such as TNF‐α, IL‐1β and IL‐6. Its antioxidant effects, mediated in part through activation of the Nrf2/HO‐1 pathway, further protect retinal cells from oxidative stress, a key driver of both vascular and inflammatory damage under hypoxic conditions [8, 9]. These pleiotropic mechanisms position RSV as an attractive dual‐function therapeutic for retinal neovascular diseases, offering the potential to concurrently suppress aberrant vessel growth and mitigate inflammation. Despite its promising therapeutic properties, RSV's clinical application is hindered by its poor solubility, low bioavailability and instability under physiological conditions [10]. Therefore, it is crucial to optimise the solubility, bioavailability and stability of RSV for its effective application.

Tetrahedral framework nucleic acids (tFNAs) have emerged as a cutting‐edge biomolecular nanomaterial, generating significant interest due to their remarkable capabilities in drug delivery systems [11]. Unlike traditional carriers such as liposomes and PLGA/poly (lactic acid) nanoparticles [12], tFNAs are distinguished by their simple synthesis, excellent biocompatibility and enhanced tissue permeability. Research has demonstrated the effectiveness of tFNAs in transporting various therapeutic agents, including oligonucleotides (e.g., siRNA, miRNA), peptides (notably antimicrobial peptides) and small molecules (e.g., RSV) [13, 14, 15]. Importantly, the anti‐inflammatory and antioxidant properties of tFNAs stem from their ability to modulate the MAPK and NF‐κB signalling pathways, like the mechanisms of RSV [16]. This combination indicates that a strategy incorporating both tFNAs and RSV could address the limitations of RSV when used alone, thus improving anti‐inflammatory and antioxidant effects.

Here, we developed a tFNAs–RSV nanocomplex designed to improve RSV stability, solubility and intraocular delivery efficiency. We hypothesised that this platform would exert dual anti‐angiogenic and anti‐inflammatory effects and protect retinal function in neovascular disease. This study evaluates the physicochemical properties, in vitro efficacy, in vivo therapeutic performance, and mechanistic underpinnings of tFNAs–RSV, aiming to establish a versatile nanoplatform for precision treatment of retinal neovascular disorders.

## 2 Results

### 2.1 Synthesis and Characterisation of tFNAs‐RSV

**FIGURE 1:**
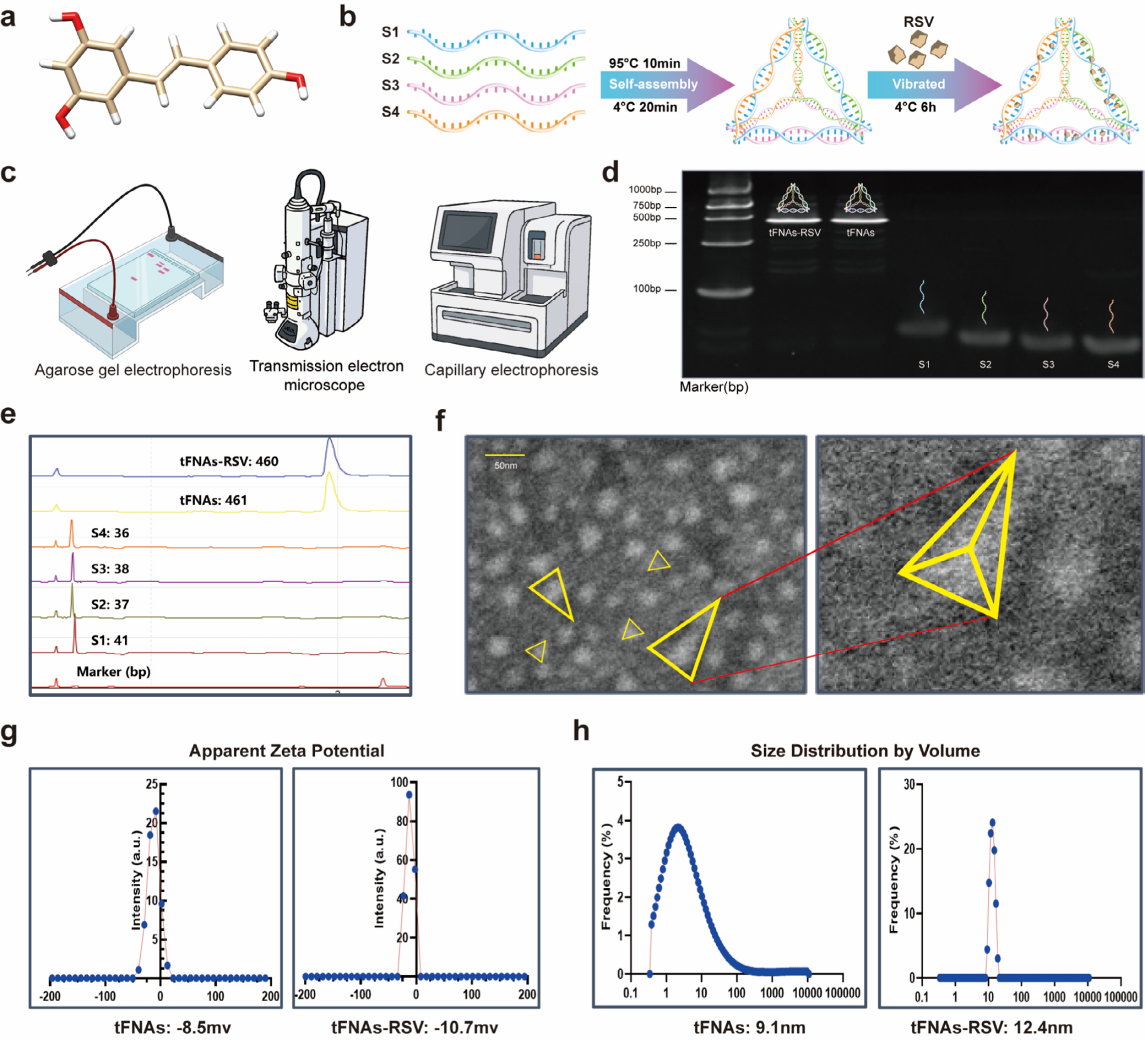
The synthesis and physicochemical properties of tFNAs‐RSV.(a) The chemical structure of Resveratrol (RSV). (b) Schematic diagram of the synthesis of tFNAs‐RSV. (c) Schematic diagram of the agarose gel electrophoresis, transmission electron microscope, and capillary electrophoresis. (d) The weights of nucleotide single strands, tFNAs and tFNAs‐RSV measured by polyacrylamide gel electrophoresis (PAGE). (e) The weights measured by high‐performance capillary electrophoresis (HPCE). (f) Transmission Electron Microscope (TEM) image of the synthesised tFNAs‐RSV. Scale bars are 50 nm. (g) Stability of tFNAs‐RSV was measured by zeta potential analysis. (h) The larger size of tFNAs‐RSV than tFNAs was detected by dynamic light scattering (DLS).

The chemical structure of RSV, a small‐molecule polyphenol, is shown in Figure 1a. The tFNAs‐RSV complex was synthesised following the flowchart presented in Figure 1b. The synthesis process began with the design of four specific single‐stranded DNA sequences capable of self‐assembling into tFNAs under appropriate conditions. Subsequently, the assembled tFNAs were mixed with RSV and incubated with gentle shaking at 4°C for 6 h, allowing RSV to adsorb onto and bind to the tFNA structure. The successful synthesis of tFNAs and the tFNAs‐RSV complex was confirmed by 8% polyacrylamide gel electrophoresis (PAGE), with results matching the theoretical structure of tFNAs formed by four single‐stranded DNA molecules, as documented in earlier studies (Figure 1c,d). High‐performance capillary electrophoresis (HPCE) further validated these findings, yielding consistent results that aligned with those observed in PAGE (Figure 1e). The transmission electron microscopy (TEM) analysis confirmed the triangular morphology of tFNAs‐RSV, consistent with previously reported structures (Figure 1f). Measurements of Zeta potential indicated values of −8.5 mV for tFNAs and −10.7 mV for tFNAs‐RSV (Figure 1g), indicating the stability of the complexes. Dynamic light scattering (DLS) analysis revealed particle sizes of 9.1 nm for tFNAs and 12.4 nm for tFNAs‐RSV, demonstrating that RSV incorporation caused a slight increase in size while maintaining structural integrity (Figure 1h).

### 2.2 tFNAs‐RSV Inhibits Endothelial Cell Proliferation, Migration and Tube Formation In Vitro

**FIGURE 2:**
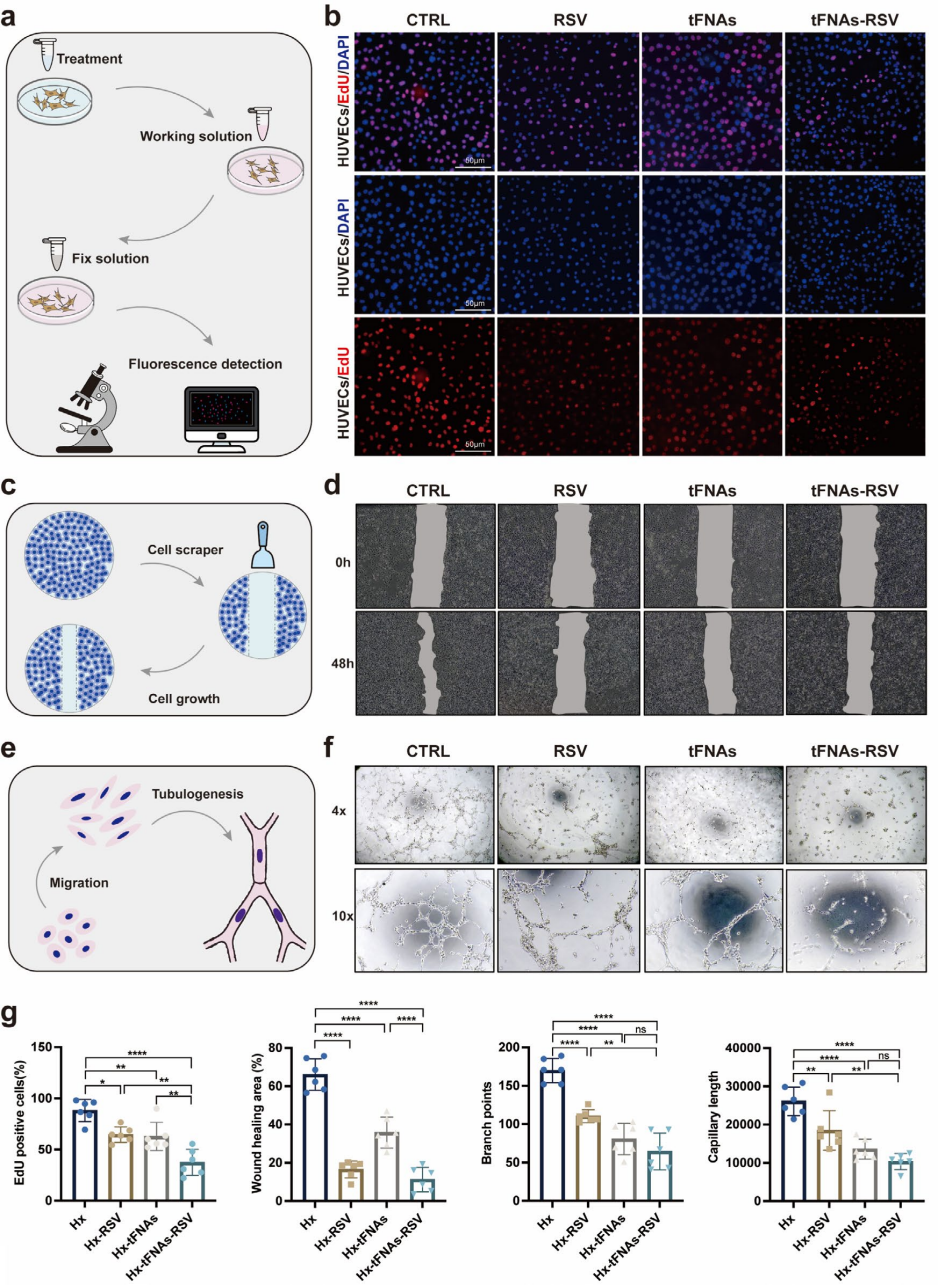
tFNAs‐RSV inhibits endothelial cell proliferation, migration, and tube formation in vitro. (a) Schematic diagram of the EdU assay. (b) Results of the cell proliferation assay of HUVECs following different treatments, along with analysis of the ratio of positive nuclei colocalised with EdU (red) to Hoechst (blue). Scale bars represent 50 μm. (c) Schematic diagram of the scratch‐wound assay. (d) Binary image results of scratch‐wound assay on HUVECs at 0 and 48 h after different treatments and analysis of wound healing area (presented as ratio of 48 h to 0 h). Scale bars are 500 μm. (e) Schematic diagram of the scratch‐wound assay. (f) The results of tube formations of HUVECs after different treatments and analysis of branch points and capillary lengths. (g) The statistical results for the three in vitro experiments. Data are presented as mean ± SD (*n* = 6). RSV, Resveratrol; tFNAs, tetrahedral framework nucleic acids; tFNAs‐RSV, Tetrahedral framework nucleic acids combined with Resveratrol; HUVECs, human umbilical vein endothelial cells. Statistical analysis: ANOVA test was applied, *p < 0.05, **p < 0.01, ***p < 0.001, **** *p* < 0.0001. All abbreviations and *p*‐values are applied throughout the text without further notation.

Under hypoxia conditions, HUVECs were divided into four treatment groups: vehicle control, RSV (80 μM), tFNAs (250 nM) and tFNAs‐RSV (tFNAs: 250 nM, RSV: 80 μM). After 24 h of incubation under hypoxic (37°C, 1% O_2_, 5% CO₂), the EdU assay (Figure 2a,b) showed that tFNAs‐RSV significantly reduced cell proliferation (37.52% ± 12.83%) compared to the vehicle control (88.14% ± 10.72%, *p* < 0.0001). Proliferation in the RSV (64.71% ± 12.83%, *p* = 0.0103) and tFNAs (62.87% ± 13.83%, *p* = 0.0054) groups was also reduced, though to a lesser degree. Notably, tFNAs‐RSV was significantly more effective than either RSV or tFNAs alone in mitigating hypoxia‐induced proliferation (*p* = 0.0028 and *p* = 0.0053, respectively). As shown in Figure 2c, tFNAs‐RSV significantly inhibited HUVEC migration (11.33% ± 6.36%), compared to the vehicle control (66.05% ± 8.23%, *p* < 0.0001) and the tFNAs‐treated group (35.85% ± 8.12%, *p* < 0.0001). Migration rates between RSV and tFNAs‐RSV were not significantly different (16.59% ± 4.40% vs. 11.33% ± 6.36%, *p* = 0.6829), suggesting comparable efficacy in reducing cell motility (Figure 2d). The tube formation assay (Figure 2e) further illustrated the anti‐angiogenic effects of tFNAs‐RSV. The number of branch points in the tFNAs‐RSV group (64.50 ± 23.90) was significantly lower than that in vehicle controls (170.0 ± 15.72, *p* < 0.0001). While the reductions observed in the RSV (110.5 ± 8.48/field) and tFNAs (80.50 ± 20.48/field) groups were noteworthy, they were not significantly different from those in the tFNAs‐RSV group (*p* = 0.5331). Regarding capillary length, tFNAs‐RSV outperformed RSV (*p* = 0.0045) but exhibited comparable results to tFNAs (*p* = 0.5235) (Figure 2f). The statistical results for the three in vitro experiments were shown in Figure 2g. These findings collectively highlight the superior performance of tFNAs‐RSV in suppressing key angiogenic processes in vitro, including proliferation, migration, and tube formation, under hypoxic conditions. The enhanced efficacy of tFNAs‐RSV in reducing cell proliferation compared to RSV and tFNAs alone suggests a synergistic effect arising from the combination of tFNAs' drug delivery capabilities and RSV's bioactive properties. By stabilisng RSV and enhancing its cellular uptake, tFNAs provide a platform that maximises the therapeutic potential of RSV.

### 2.3 tFNAs‐RSV Suppresses Pathological Angiogenesis and Improves Vascular Microstructure In Vivo

**FIGURE 3:**
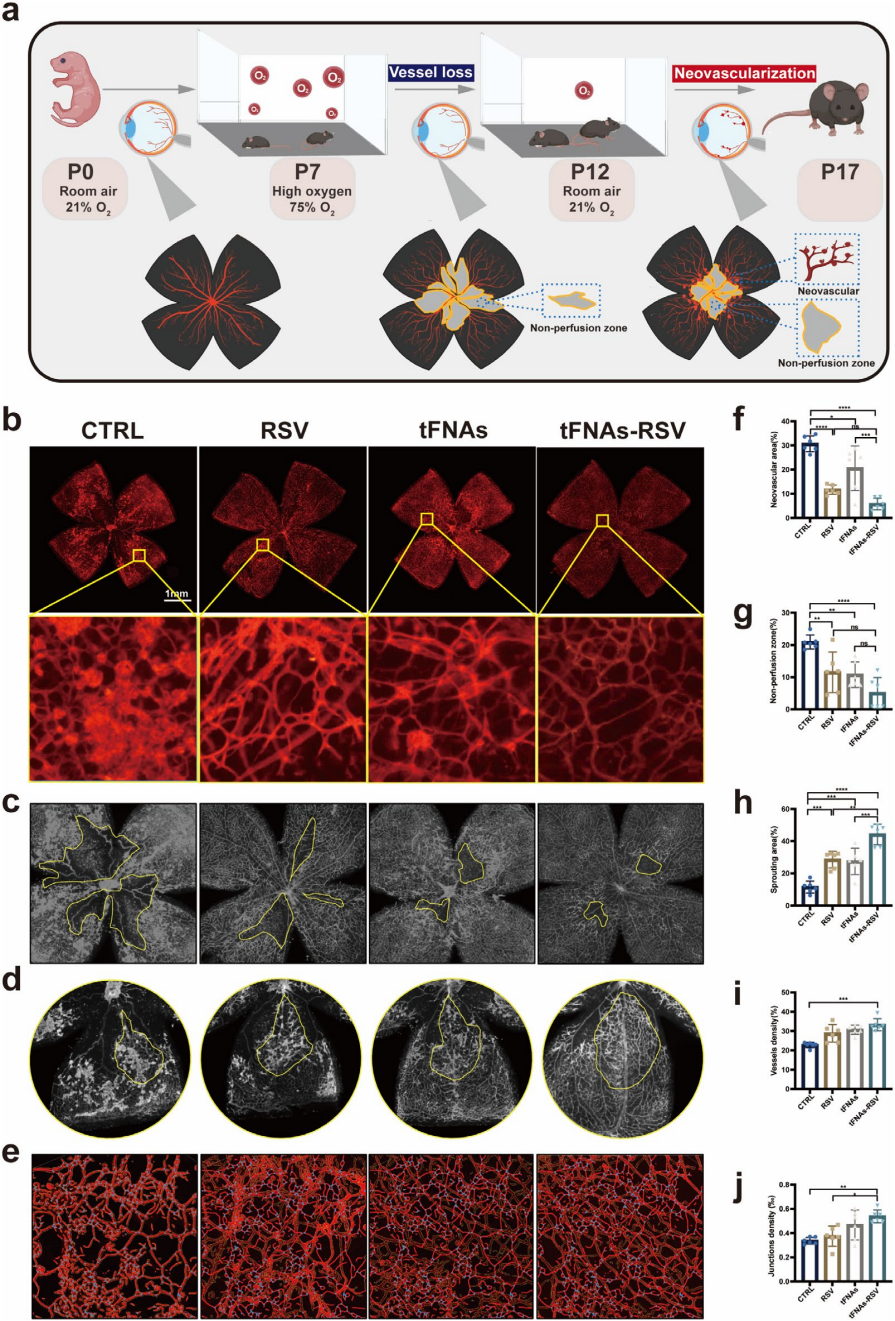
tFNAs‐RSV inhibit pathological angiogenesis and reduce avascular area in OIR model on retinal flat‐mount.(a) Schematic diagram of tFNAs‐RSV administration in OIR mouse models. (b) Analysis of neovascularisation of the retina mount, stained by IB4. (c) Local magnification diagram of retina mount and analysis of neovascular area. (d) The vaso‐obliterated area (VO) is portrayed by a yellow line in the OIR model and analysis of the non‐perfusion zone. (e) The image of higher magnification of vessels sprouting from the vein and the sprouting area of each group. (f) Quantitative analysis of the neovascular area. (g) Non‐perfusion zone. (h) Sprouting area. (i) Vesseles density. (j) Junction density.

The oxygen‐induced retinopathy (OIR) model in C57BL/6J mice was employed to evaluate the therapeutic potential of tFNAs‐RSV. On postnatal Day 12 (P12), C57BL/6J pups received intravitreal injections of vehicle control, RSV (80 μM), tFNAs (250 nM) or tFNAs‐RSV (tFNAs: 250 nM, RSV: 80 μM). Retinas were harvested on P17 and prepared as flat mounts stained with IB4 to assess retinal neovascularisation and non‐perfusion area (Figure 3a). Vehicle‐treated OIR mice exhibited significant neovascular areas (NVA, 30.67% ± 3.27%) and non‐perfusion areas (NPA, 20.91% ± 2.17%), confirming the successful establishment of the OIR model (Figure 3b,c). Both RSV and tFNAs treatments reduced NVA to 11.68% ± 1.89% and 20.58% ± 9.21%, respectively. Notably, tFNAs‐RSV significantly inhibited NVA, reducing it to 5.79% ± 2.44% (*p* < 0.0001), demonstrating superior therapeutic efficacy (Figure 3b,f). tFNAs‐RSV also decreased NPA to 5.09% ± 4.81% (*p* < 0.0001) compared to the vehicle control group (Figure 3c,g). In addition, the sprouting area from veins was significantly higher in the tFNAs‐RSV group (44.21% ± 6.38%) compared to the control group (11.49% ± 3.72%, *p* < 0.0001) and other treatment groups (Figure 3d,h). Enhanced vascular density (33.26% ± 3.14% vs. 22.78% ± 1.39%, *p* = 0.0004) and junction density (0.54 ± 0.05 vs. 0.33 ± 0.03, *p* = 0.0016) further highlight the ability of tFNAs‐RSV to not only reduce pathological neovascularisation but also improve the retinal vascular structure (Figure 3e,i,j).

### 2.4 tFNAs‐RSV Inhibit Retinal Inflammation Induced by Hypoxia

**FIGURE 4:**
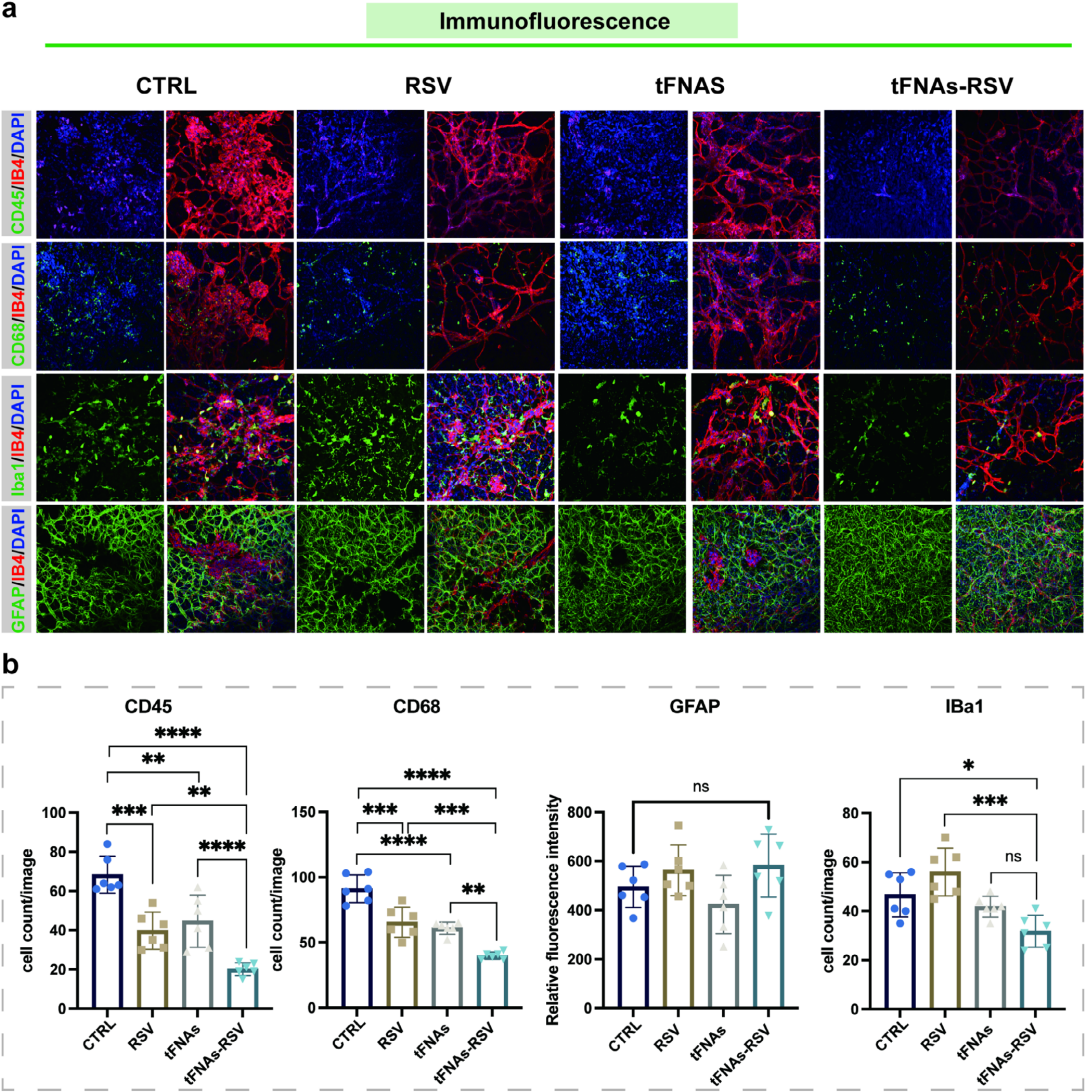
tFNAs‐RSV inhibit retinal inflammation. (a) Retinal mount from different groups showing CD45^+^ (purple), CD68^+^ (green), Iba1^+^ microglia (green) and GFAP^+^ astrocytes (green) cells, with analysis results. (b) OIR, oxygen‐induced retinopathy; tFNAs‐RSV, Tetrahedral framework nucleic acids combined with Resveratrol; RSV, Resveratrol; tFNAs, tetrahedral framework nucleic acids. Statistical analysis: Data are presented as mean ± SD, one‐way ANOVA, (*n* = 6) * *p* < 0.05, **p < 0.01, ***p < 0.001, ****p < 0.0001.

Building on this, we further investigated the inflammatory response in retinal tissue (Figure 4a,b). Specifically, we quantified CD45^+^ and CD68^+^ cells after tFNAs‐RSV treatment. The vehicle control group exhibited 68.33 ± 9.44 CD45^+^ cells and 91.17 ± 10.65 CD68^+^ cells, which were significantly reduced to 20.17 ± 3.25 and 39.83 ± 2.71, respectively, following tFNAs‐RSV treatment. The reduction of CD45^+^ and CD68^+^ cells, which mark leukocyte infiltration and macrophage activation, respectively, suggests a significant attenuation of the inflammatory response in retinal tissues treated with tFNAs‐RSV. Moreover, the tFNAs‐RSV outperformed the RSV and tFNAs groups in reducing inflammatory cell counts. Activated microglia (Iba1^+^) were also reduced in the tFNAs‐RSV group compared to controls (31.83 ± 6.55 vs. 46.67 ± 9.05, *p* = 0.0167), while RSV treatment increased microglial activation (56.00 ± 9.78). Surprisingly, GFAP^+^ astrocyte activity remained unchanged. No significant differences were observed in astrocyte (GFAP^+^) expression among the groups (*p* = 0.0841). The absence of significant changes in GFAP^+^ expression, a marker of astrocyte activation, indicates that tFNAs‐RSV selectively targets microglial‐mediated inflammation and angiogenesis without broadly affecting glial activation.

### 2.5 tFNAs‐RSV Treatment Preserves Retinal Electrophysiological Function In Vivo

**FIGURE 5:**
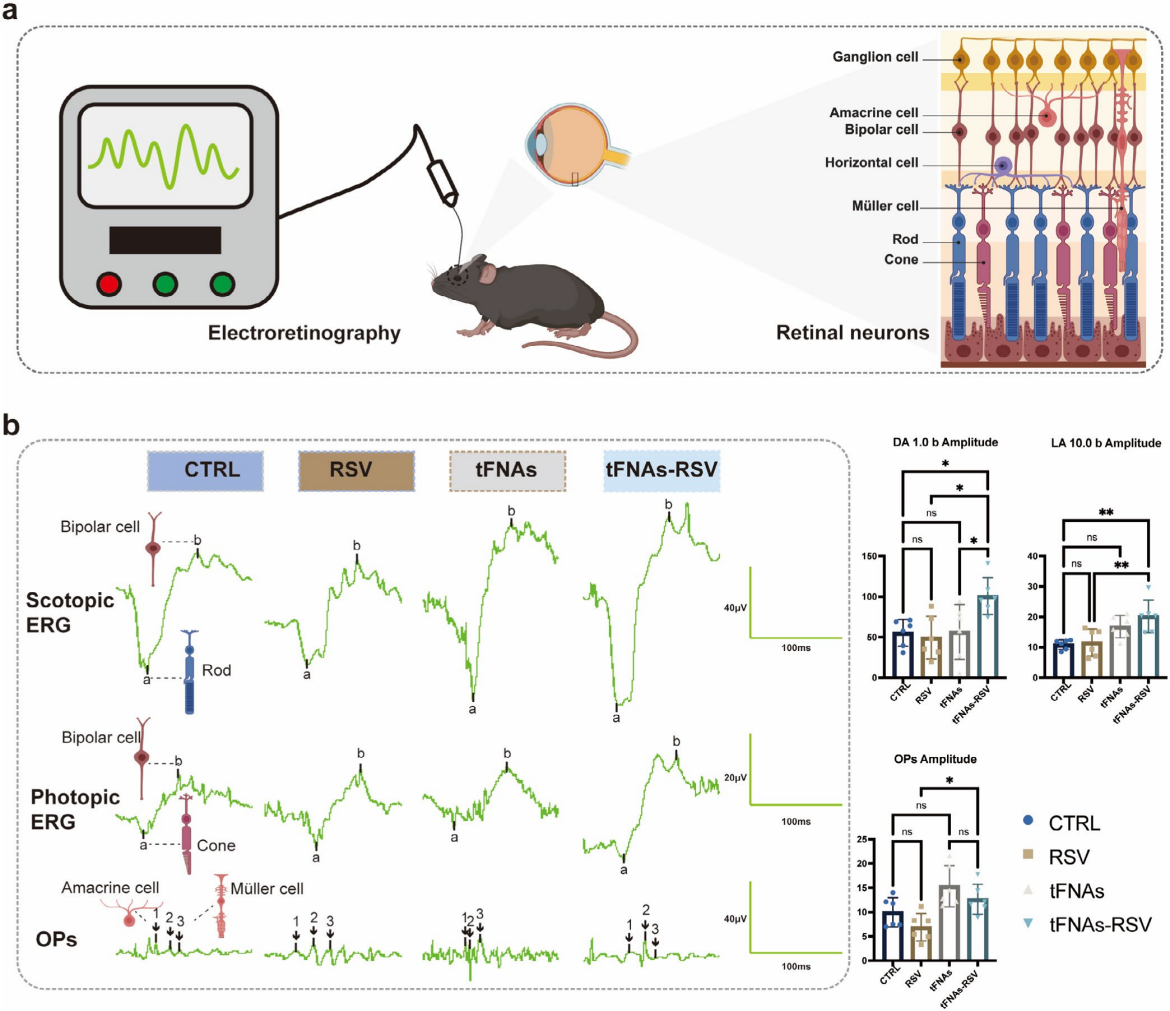
tFNAs‐RSV preserves retinal electrophysiological function. (a) Retinal function in mice with different treatments was assessed using scotopic and photopic ERGs on P17. (b) Scotopic ERG (1.0 cd s m^−2^), photopic ERG (10.0 cd s m^−2^), and SOPs showed a higher amplitude than the CTRL group. ERG, electroretinogram; OPs, oscillatory potentials; SOPs, summed Ops. Statistical analysis: Data are presented as mean ± SD, one‐way ANOVA (*n* = 6)

The preservation of retinal electrophysiological function is essential for maintaining visual clarity and overall vision quality. To assess the impact of tFNAs‐RSV on retinal function, we performed flash ERG (fERG) analysis on P17 OIR mice, focusing on scotopic (rod), photopic (cone) and oscillatory potentials (OPs) (Figure 5a). Implicit times did not differ significantly across groups (*p* > 0.05, Figure 5b). However, in the scotopic b‐wave, the tFNAs‐RSV group showed significantly higher amplitudes than the control group, RSV group and tFNAs group. In the photopic b‐wave, the tFNAs‐RSV group significantly surpassed those of the control and RSV groups, though it did not differ significantly from the tFNAs group. Regarding OPs, the tFNAs‐RSV group recorded a higher average amplitude than that of the RSV group. These findings suggest that tFNAs‐RSV exerts potent neuroprotective effects, which may delay or repair neuronal damage caused by ischemic retinal diseases, thereby improving visual function.

### 2.6 RNA‐Seq Reveals tFNAs‐RSV Inhibits Neovascularisation Primarily by Regulating the MAPK and HIF‐1α Pathway, and Inhibits Inflammation by Down‐Regulating the NF‐κB Pathway

**FIGURE 6:**
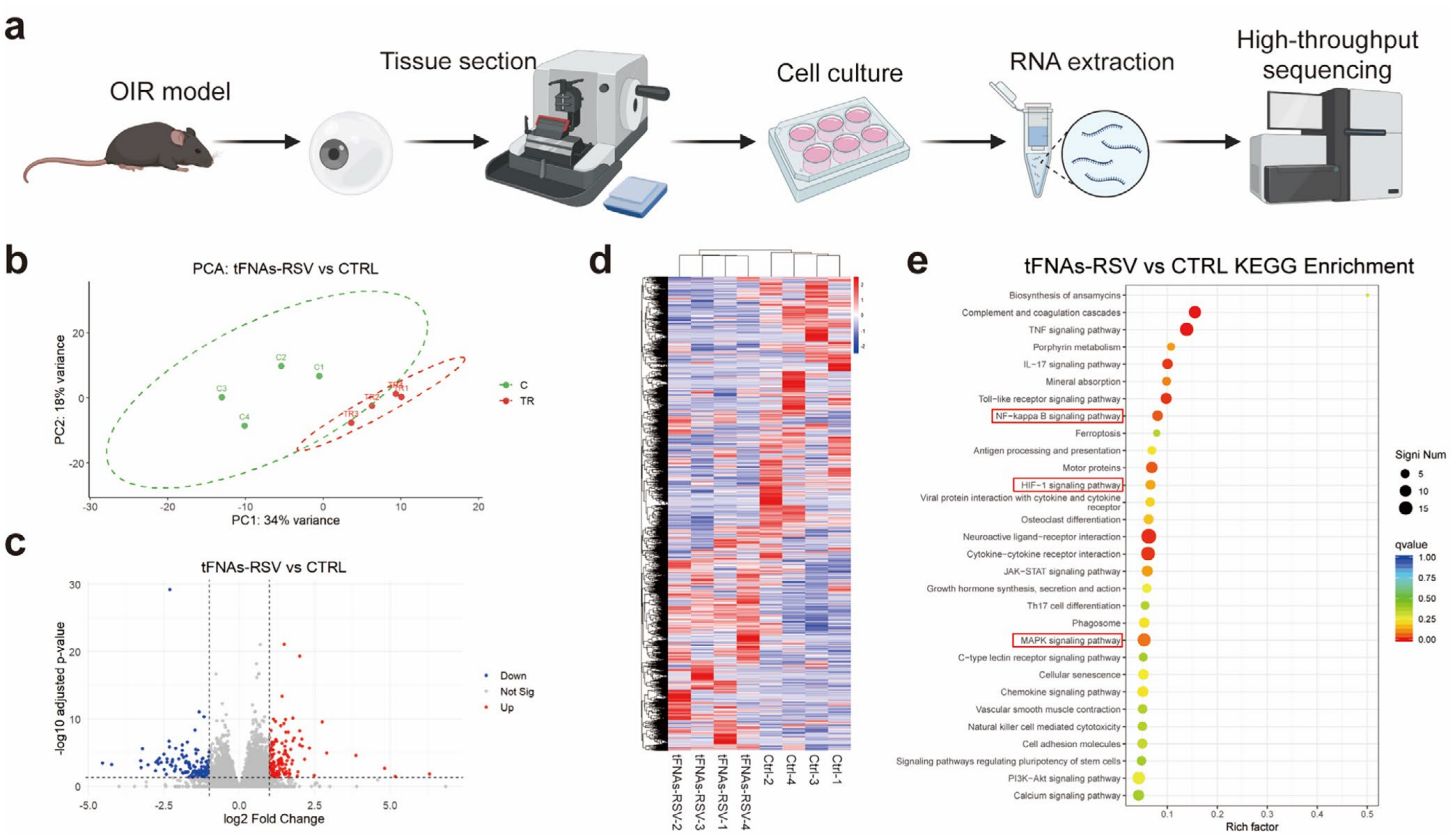
RNA‐Seq analysis shows regulating MAPK and NF‐κB pathway after tFNAs‐RSV treatment.(a) Schematic representation of the RNA sequence of the C57BL/6 mouse OIR model. (b) (PCA) showed a clear separation between the tFNAs‐RSV group and vehicle control. (c) Volcano plot illustrating changes in retinal RNA expression under different treatments. (d) Heatmaps comparing gene expression between the tFNAs‐RSV and RSV groups. (e) The KEGG enrichment dot plots demonstrate down‐regulation of the MAPK, HIF‐1α and NF‐κB pathways in the three comparisons.

To elucidate the molecular mechanisms by which tFNAs‐RSV inhibits neovascularisation, transcriptomic sequencing was performed on retinal tissues from the OIR model (Figure 6a). Principal component analysis (PCA) showed a clear separation between the tFNAs‐RSV group and vehicle control, indicating distinct global gene expression profiles (Figure 6b). The results revealed a total of 1829 differentially expressed genes (DEGs), which included 995 genes that were up‐regulated and 834 that were down‐regulated (Figure 6c,d). The KEGG enrichment revealed significant down‐regulation of the MAPK and HIF1α signalling pathways in the tFNAs‐RSV group relative to the control (Figure 6c,d). In addition, the NF‐κB pathways were also notably down‐regulated following tFNAs‐RSV treatment (Figure 6e). These findings highlight the MAPK/HIF‐1α pathway as a primary target of tFNAs‐RSV's anti‐neovascularisation effects, with additional modulation of NF‐κB pathways contributing to its protective impact. This multifaceted pathway regulation underscores the potential of tFNAs‐RSV as a therapeutic strategy for inhibiting pathological retinal neovascularisation.

### 2.7 tFNAs‐RSV Inhibit Hypoxia Induced Retinal Neovascularisation and Inflammation via Down‐Regulating MAPK and NF‐κB Pathway

**FIGURE 7:**
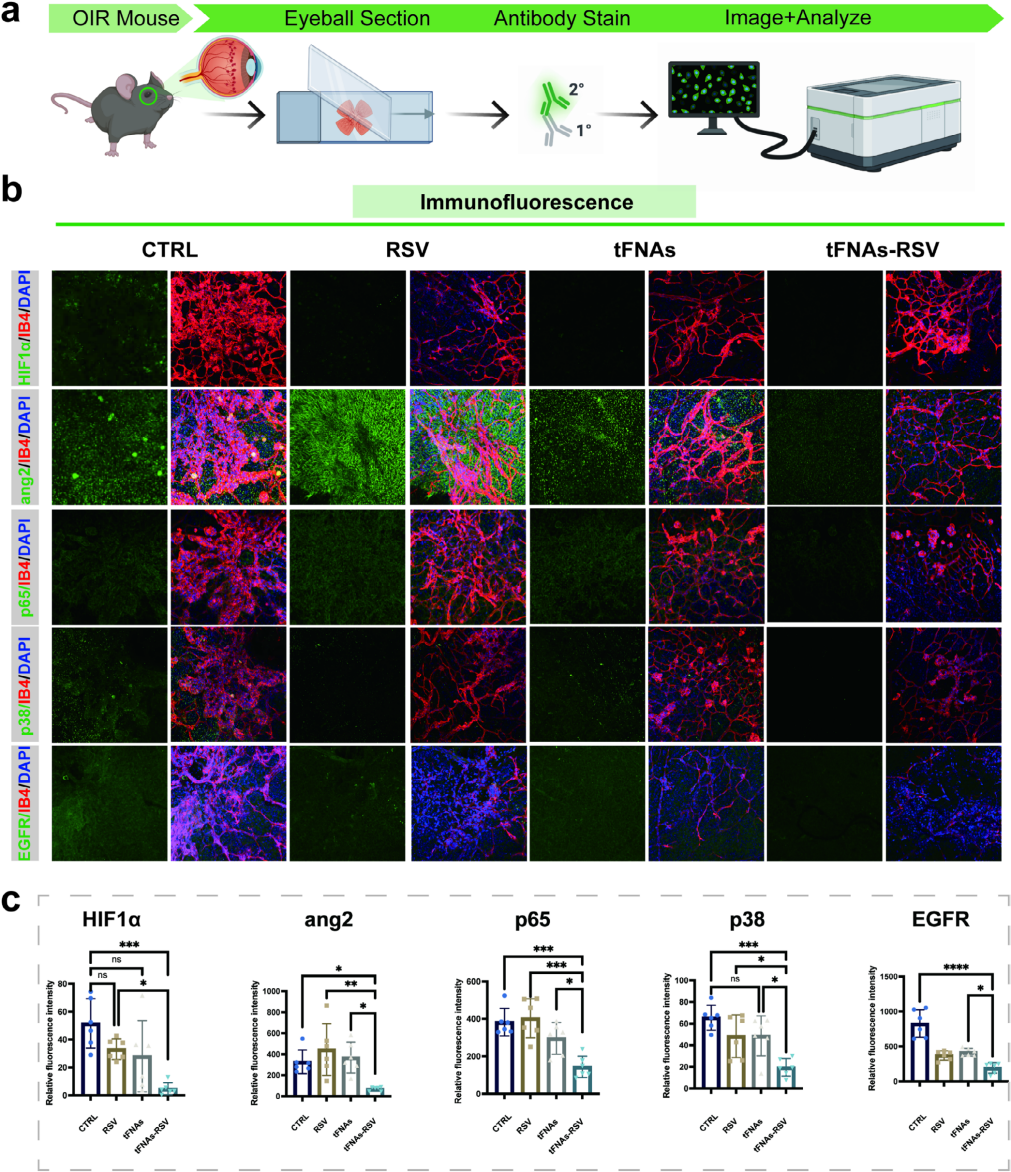
tFNAs‐RSV inhibit retinal neovascularisation via down‐regulating p38 MAPK and NF‐κB p65 pathway. (a) The schematic of immunofluorescence staining in a retinal whole‐mount. (b) Immunofluorescence staining of the retinal mounts also displays HIF1α, Ang‐2, p65, p38 and EGFR (green) expression in the tFNAs‐RSV group compared to other groups. (c) Statistical analysis shows the expression of five proteins is significantly reduced in the tFNAs‐RSV group. Statistical analysis: Data are presented as mean ± SD, one‐way ANOVA, (*n* = 6). *p < 0.05, **p < 0.01, ***p < 0.001, ****p < 0.0001.

To validate the RNA‐Seq findings, immunofluorescence staining was conducted on retinal flat mounts from P17 OIR mice to examine the expression of key pathway components (Figure 7a). HIF‐1α, a critical regulator of hypoxic responses, induces the expression of pro‐angiogenic factors such as Ang2, which destabilises vascular integrity and enhances endothelial responsiveness to VEGF, thus exacerbating neovascularisation. The expression of HIF‐1α was highest in the control group, moderately reduced in the RSV and tFNAs groups, and significantly decreased in the tFNAs‐RSV group (*p* = 0.0003). Ang2 showed no significant reduction in the RSV or tFNAs groups, but a notable decrease was observed in the tFNAs‐RSV group compared to controls (*p* = 0.0446). The pro‐inflammatory mediators p65 (a subunit of NF‐κB) and p38 (a component of the MAPK pathway) amplifies the inflammatory response under hypoxia, fueling the production of cytokines and worsening vascular pathology. The expression levels of p65 were significantly lower in the tFNAs‐RSV group compared to all other groups (*p* < 0.05). Similarly, p38 expression was markedly down‐regulated in the tFNAs‐RSV group compared to the control (*p* = 0.0002), RSV (*p* = 0.0184) and tFNAs groups (*p* = 0.0170). Additionally, EGFR expression was significantly reduced in the tFNAs‐RSV group compared to controls (*p* < 0.0001) and the tFNAs group (*p* = 0.0134), but not in the RSV group (Figure 7b,c). The observed down‐regulation of HIF‐1α, Ang2, p65, p38 and EGFR in the tFNAs‐RSV group highlights the complex interplay between angiogenesis and inflammation in the pathological progression of retinal neovascularisation.

Based on the mechanistic findings, these results demonstrate that tFNAs‐RSV exerts anti‐angiogenic effects mediated by suppression of the HIF‐1α/Ang‐2 signalling axis and MAPK pathway and anti‐inflammatory effects achieved through inhibition of the NF‐κB pathway, thereby collectively attenuating hypoxia‐triggered neovascularisation and inflammatory responses.

## 3 Discussion

Retinal neovascular diseases remain a global challenge, often culminating in severe and irreversible vision loss. Pathological neovascularisation is the main pathogenic mechanism of the disease, while the inflammatory response is also an important factor that cannot be ignored [17, 18]. Current treatments, such as anti‐VEGF therapy and laser therapy, focus more on retinal ischemia and the inhibition of neovascularisation [19, 20]. Our design simultaneously considers both anti‐angiogenic and anti‐inflammatory effects, thereby exerting a dual role in inhibiting retinal neovascularisation and protecting retinal nerves.

RSV has shown promise in preclinical studies as an anti‐angiogenic agent [21, 22, 23]. For instance, intravitreal injection of RSV effectively suppressed laser‐induced choroidal neovascularisation [21]. However, its poor bioavailability, instability under physiological conditions, and limited tissue penetration significantly hinder its clinical utility. These limitations necessitated the development of a delivery platform that enhances the therapeutic potential of RSV while addressing these pharmacological barriers. In our previous studies, tFNAs have been proven to be an excellent delivery system, and they themselves also possess a certain effect in inhibiting neovascularisation. In the present study, we combined RSV with tFNAs to enhance delivery efficiency, stability and efficacy [11]. Using the C57BL/6J OIR model, we demonstrated that intravitreal injection of tFNAs‐RSV not only significantly inhibited pathological retinal neovascularisation but also unexpectedly facilitated vascular remodelling. This remodelling was characterised by a reduction in avascular zones, increased vascular density and enhanced junction density. Notably, such remodelling of pathological vessels represents an efficacy not exhibited by current anti‐VEGF drugs.

Given that RSV itself has anti‐inflammatory properties, we further examined whether tFNA‐RSV exerts similar anti‐inflammatory effects on ischemia‐induced inflammation. After treatment with tFNA‐RSV, the numbers of T cells, macrophages and microglia cells induced by ischemia were all reduced, indicating that the ischemia‐induced inflammatory cell infiltration was significantly inhibited. Moreover, such effects, including the inhibition of neovascularisation, remodelling of pathological blood vessels, and regulation of inflammatory cells, protected the retinal neurovascular unit, which was manifested by more active ERG waveforms [24, 25, 26]. The mechanisms underlying this dual action likely stem from the dual anti‐inflammatory and anti‐angiogenic properties of RSV, amplified by the delivery capabilities of tFNAs.

RNA sequencing results and further experimental verification likewise explain the dual effects of tFNAs‐RSV. We observed significant regulation of the p38 MAPK [27], HIF‐1α [28] and NF‐κB p65 [29] pathways, which are central to the crosstalk between inflammation and angiogenesis. Examination of retinal flat mounts from OIR mice revealed that HIF1α, ANG‐2, P65, P38 and EGFR were all down‐regulated. These proteins correspond to key components of the HIF1α, NF‐κB p65 and p38 MAPK pathways, respectively. Regulating multiple pathways may confirm a major advantage of tFNAs‐RSV.

This study provides compelling preclinical evidence that tFNAs‐RSV can concurrently suppress hypoxia‐induced angiogenesis and inflammation in the retina. However, several limitations should be acknowledged. First, the therapeutic evaluation was conducted exclusively in rodent models of retinal neovascularisation, which, while well established, may not fully recapitulate the complexity of human retinal vascular diseases. Second, the follow‐up period was relatively short, and long‐term efficacy, safety, and potential immune responses to repeated administration remain to be determined. Third, the pharmacokinetics and biodistribution of tFNAs‐RSV in larger eyes, as well as its ability to penetrate human ocular barriers, require further investigation to support clinical translation. Fourth, although the study elucidated key anti‐inflammatory and anti‐angiogenic mechanisms, the broader transcriptomic and proteomic impacts of RSV delivered via tFNAs under chronic disease conditions were not explored. Finally, the scalability, stability and cost‐effectiveness of large‐scale tFNA‐RSV production must be addressed before advancing toward clinical application.

## 4 Conclusion

In this study, we demonstrate the therapeutic promise of tFNAs‐RSV for retinal neovascular diseases. By concurrently targeting the p38 MAPK and NF‐κB p65 pathways, tFNAs‐RSV suppresses pathological angiogenesis, mitigates inflammation, promotes vascular remodelling and preserves retinal integrity. Its enhanced stability and delivery efficiency address key limitations of conventional treatments, providing a comprehensive strategy for disease management. Future investigations should assess its long‐term impact on visual function and refine its application across disease stages, paving the way for its translation into a next‐generation, dual‐action therapy that redefines the treatment paradigm for retinal vascular diseases.

## 5 Methods

### 5.1 Synthesis and Characterisation of tFNAs‐RSV

Four designed assembled ssDNA were synthesised as described before [14]. Assembled tFNAs were concentrated with a DNA PacPA100 (Thermo Scientific, USA) chromatographic column with HPLC at 1 mL min^−1^. Different mobile phases were used (mobile phase A: 25 mM Tris–HCL, mobile phase B: 25 mM Tris–HCL + 375 mM NaClO_4_). Subsequently, RSV (80 μM) was mixed with tFNAs (250 nM) by vibrating for 6 h at 4°C^12^. The concentration ratio of RSV (80 μM) to tFNAs (250 nM) was selected based on previous studies [30] demonstrating optimal loading efficiency and structural stability of tFNAs‐RSV nanocomplexes under this condition. Unloaded RSV and remaining ssDNA were eliminated after ultrafiltration (30 kDa). We use PAGE, HPCE, zeta potentials, DLS and TEM to verify the successful synthesis and characterisation of tFNAs‐RSV.

### 5.2 Cell Culture, Treatment and Angiogenesis Experiments

HUVECs (from ATCC) were incubated with DMEM/F‐12 medium (Gibco, USA) supplemented with 10% foetal bovine serum (Gibco, USA), and 1% antibiotic solution (Gibco, USA) under a normoxic environment (37°C, 95% air, 5% CO_2_) for 24 h. Next, HUVECs were cultured under hypoxia (37°C, 1% O_2_, 5% CO_2_) with various treatments (control, 250 nM tFNAs, 80 μM RSV or tFNAs‐RSV [tFNAs: 250 nM, RSV: 80 μM]) followed by 24 h of re‐oxygenation in regular medium. Briefly, the EdU cell proliferation assay was conducted using Click‐iT EdU Imaging Kits (Beyotime, China); the wound healing experiment was performed using the cell migration assay [14], and the tube formation assay was conducted with Matrigel solution (50 μL per well). The images were quantified using the Angiogenesis Analyser plugin for ImageJ.

### 5.3 OIR Model, Intravitreal Injection

All animal studies were approved by the Institutional Animal Care and Use Committee of Zhongshan Ophthalmic Center (Z2023058). OIR was carried out in C57BL/6J mice as previously described [14, 15]. At P12, the mice were anaesthetised with 1% pentobarbital sodium (50 mg kg^−1^). 1 μL of vehicle, tFNAs (250 nM) or RSV (80 μM) or tFNAs‐RSV (250 nM:80 μM) was injected intravitreously using a 33‐gauge syringe (Hamilton, USA).

### 5.4 Retinal Mounts and Immunofluorescence

Mice at P17 were euthanised, and their eyes were enucleated and fixed in freshly prepared 4% paraformaldehyde (PFA) for 1 h. Retinas were carefully dissected under a stereo microscope (MZ62, Mshot, Guangdong, China). The isolated retinas were subsequently blocked and permeabilised in PBS containing 1% bovine serum albumin (BSA) and 0.5% Triton X‐100. Samples were then incubated overnight at 4°C with different primary antibodies in the following: HIF‐1α (1:200, 36169S, CST), IB4 (1:200, I21411, ThermoFisher), ang‐2 (1:250, ab8452, Abcam), CD68 (1:800, 97778S, CST), CD45 (1:100, 550,539, BD), EGFR (1:100, 4267S, CST), GFAP (1:400, 80788S, CST), Iba1 (1:1000, ab178846, Abcam), p38 (1:100, YT3513, Immunway), p65 (1:1000, GB11997‐100, Servicebin), p16 (1:100, ab51243, Abcam). Following incubation, retinas were thoroughly washed in PBS, mounted onto slides, and examined using an inverted microscope (Ts2FL, Nikon, Japan).

### 5.5 RNA Sequencing

Dissect the eyeballs of P17 pups in an ice‐cold environment to isolate the retina. Transfer the isolated retinas into RNase‐free tubes containing RNA stabilisation reagent and store at −80°C. Extract total RNA following the manufacturer's protocol. Assess RNA quantity and purity using a spectrophotometer and integrity via an RNA bioanalyzer. Retain samples with an RNA integrity number (RIN) ≥ 7 for sequencing. Use a poly(A)‐enrichment or ribosomal RNA depletion strategy for mRNA isolation [31]. Construct libraries using a commercially available RNA sequencing kit. Adhere to the protocol, including fragmentation, cDNA synthesis, end repair, adapter ligation and amplification. Perform library quality control using a bioanalyzer and quantify with a fluorometer. Pool libraries equimolarly and sequence on a high‐throughput platform with paired‐end 150 bp reads. Target a read depth of at least 20 million reads per sample. Perform quality control using tools such as FastQC [32]. Align reads to the mouse genome using STAR. Quantify gene expression with featureCounts or similar software. Normalise and analyse differential expression using DESeq2 [33].

### 5.6 Electroretinogram (ERG) Assessment

To provide more accurate mice ERG parameters, the retinal function on P17 was assessed with Celeris D430 System (Diagnosys LLC, USA). The a‐wave and b‐wave amplitudes and implicit times were measured according to the International Society for Clinical Electrophysiology of Vision Standard [34, 35].

### 5.7 Statistical Analysis

Statistical analysis was performed using GraphPad Prism 9. Data are presented as the means ± SD. The number of replicates and/or the total number of animals is shown in the figure legends or within the figures. Data distribution was assessed using the Shapiro–Wilk test, and homogeneity of variance was evaluated using Levene's test before performing one‐way ANOVA. Significant differences were evaluated by ANOVA, and *p* < 0.05 was considered statistically significant.

## Author Contributions

Y.J., T.C. and J.H. contributed equally to this work. Y.W. and X.D. conceived this research. Y.J., T.C. and J.H. designed the research, collected and analysed the data, wrote the manuscript and Figures. L.C, Y.L. and J.L. provided help during data collection and analysis.

## Funding

This work was supported by the National Natural Science Foundation of China, 82271092, 82571230.

## Conflicts of Interest

The authors declare no conflicts of interest.

## Data Availability

The data that support the findings of this study are available from the corresponding author upon reasonable request.
